# The Allosteric Mechanism of G‐Protein‐Coupled Receptors is Induced Fit, Not Conformational Selection

**DOI:** 10.1002/bies.70082

**Published:** 2025-10-18

**Authors:** Kazem Asadollahi, Paul R. Gooley, Thomas R. Weikl

**Affiliations:** ^1^ Department of Biochemistry and Pharmacology Bio21 Molecular Science and Biotechnology Institute University of Melbourne Parkville Victoria Australia; ^2^ Department of Biomolecular Systems Max Planck Institute of Colloids and Interfaces Potsdam Germany

**Keywords:** binding kinetics, conformational changes, G‐protein‐coupled receptors, protein allostery, stopped‐flow mixing experiments

## Abstract

The allosteric mechanism of G‐protein‐coupled receptors (GPCRs) involves a population shift from inactive to active receptor conformations in response to the binding of ligand agonists. Two possible kinetic mechanisms for this population shift are induced fit and conformational selection. In the induced‐fit mechanism, ligands bind to inactive receptor conformations prior to the conformational transition of the receptor. In the conformational‐selection mechanism, ligands bind to active conformations after the conformational transition. For the peptide‐activated neurotensin receptor 1, stopped‐flow mixing experiments that probe the chemical relaxation into binding equilibrium and conformational transition rates measured with NMR experiments indicate an induced‐fit mechanism. For the small‐molecule‐activated $\beta_{2}$‐adrenergic receptor, an induced‐fit mechanism has been inferred from a decrease of ligand association rates after stabilization of the active receptor conformation. A structural explanation for the induced‐fit mechanism of the $\beta_{2}$‐adrenergic receptor is a closed lid over the binding site that blocks ligand entry in the active conformation. Since constriction and closing of the ligand‐binding site in the active conformation is rather common for small‐molecule‐activated and peptide‐activated GPCRs, induced fit is likely shared as allosteric mechanism by these GPCRs.

## Graphical abstract

Allosteric signaling of G‐protein‐coupled receptors (GPCRs) involves a population shift from inactive to active receptor conformations upon binding of ligand agonists. Kinetic and structural data indicate that this population shift is induced by ligand binding to inactive receptor conformations prior to the conformational change from inactive to active.

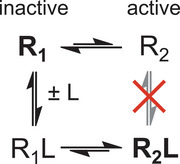

## 1 Introduction

**FIGURE 1:**
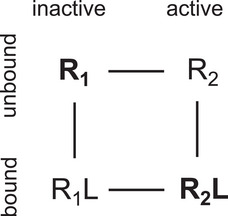
Population‐shift equilibrium model of protein allostery. In the unbound state, the inactive conformational ensemble $R_{1}$ of the protein is predominantly populated (indicated in bold), and the population of the active conformational ensemble $R_{2}$ is minor. In the ligand‐bound state, the populations are shifted, with a predominant population of $R_{2}L$ (indicated in bold) and a minor population of $R_{1}L$.

G‐protein‐coupled receptors (GPCRs) constitute the largest superfamily of cell membrane proteins and mediate the majority of cellular responses to extracellular stimuli [1]. Binding of agonist ligands to the extracellular side of GPCRs induces global conformational changes in the seven transmembrane helix domain that lead to the activation of G proteins bound to the intracellular side of GPCRs. The allosteric coupling of extracellular and intracellular binding sites of GPCRs can be understood from a chemical equilibrium of inactive and active (i.e., G‐protein‐activating) conformations that is shifted by ligand binding [2, 3] (see Figure 1). In the unbound state, the inactive conformational ensemble $R_{1}$ is predominantly populated, with a minor population of the active conformational ensemble $R_{2}$ leading to a basal, ligand‐independent level of activity. In the ligand‐bound state, the chemical equilibrium is shifted towards the active conformational ensemble $R_{2}L$.

The population shift in the equilibrium model of Figure 1 is the basis for understanding GPCR allostery, and protein allostery in general [4, 5]. But this equilibrium model does not state a kinetic sequence of events in GPCR activation. In general, two possible temporal sequences in the coupling of binding events and conformational changes of proteins are: The binding event occurs either prior to or after the conformational change [6, 7, 8, 9]. Such a clear temporal sequence of events implies that the dwell (or residence) times in the different conformational and binding states of a protein are much larger than the transition times between the states, so that a temporal overlap of conformational transitions and binding events is unlikely [10]. A sequence of events in which ligand binding occurs prior to the conformational change of a protein has been termed induced fit [11]. The opposing sequence of events in which ligand binding occurs after the conformational change of the protein is generally denoted as conformational selection [12].

However, the term conformational selection has also been used in different or broader meanings [13]. In the GPCR field, the term conformational selection has been used rather synonymously to the equilibrium concept of population shift [2, 14, 15, 16, 17, 18]. Population shift in the equilibrium model of Figure 1 is the basis for GPCR allostery, but we now ask: What is the kinetic mechanism, or the kinetic sequence of events that leads to this population shift? Does ligand‐activation of GPCRs occur via induced fit or conformational selection? In this question, the term conformational selection is used for a kinetic mechanism.

## 2 Induced Fit or Conformational Selection?

**FIGURE 2:**
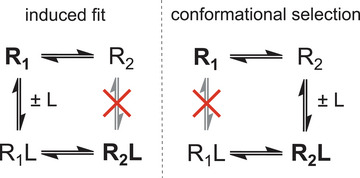
Kinetic models of protein allostery. In the induced‐fit model, only the inactive conformational ensemble $R_{1}$ of the protein is binding‐competent, and ligand binding occurs prior to the conformational change of the protein on the activation pathway from $R_{1}$ to $R_{2}L$. In the conformational‐selection model, only the active conformational ensemble $R_{2}$ is binding‐competent, and ligand binding occurs after the conformational change of the protein on the activation pathway from $R_{1}$ to $R_{2}L$.

Induced fit and conformational selection differ in the conformational ensemble in which ligand binding occurs (see Figure 2). In the induced‐fit mechanism, the ligand L binds to the inactive conformational ensemble $R_{1}$. Along the induced‐fit activation pathway from $R_{1}$ to $R_{2}L$, ligand binding therefore occurs prior to the conformational change. A conformational change from $R_{1}$ to $R_{2}$ can also occur in the unbound state, but this conformational change is “off‐pathway” to activation in the induced‐fit mechanism, that is, it does not occur along the induced‐fit activation pathway from $R_{1}$ to $R_{2}L$. In the conformational‐selection mechanism, in contrast, the ligand binds to the active conformational ensemble $R_{2}$. In this mechanism, ligand binding therefore occurs after the conformational change along the activation pathway from $R_{1}$ to $R_{2}L$. In principle, the conformational‐selection and induced‐fit pathways could also be parallel, alternative pathways. In practice, however, one of these pathways is likely the dominant pathway at the relevant ligand concentrations [19, 20], or the only possible pathway if there are structural reasons that prevent ligand binding to one of the conformational ensembles $R_{1}$ or $R_{2}$ [21].

Identifying the dominant binding mechanism requires to probe the binding kinetics. Thermodynamic principles dictate that equilibrium properties such as the equilibrium population of states are independent of the pathways along which the states can be reached. For example, the basal activity of a GPCR indicates that the active conformational ensemble $R_{2}$ is populated to some extent in the unbound state of the GPCR. However, this does not allow one to infer whether $R_{2}$ is on‐pathway (conformational selection) or off‐pathway (induced fit) regarding the activation path from $R_{1}$ to $R_{2}L$. In other words, a minor population of $R_{2}$ is a necessary condition for the conformational‐selection binding pathway on which $R_{2}$ occurs as an intermediate, but it is not a sufficient condition to infer conformational selection.

## 3 Distinguishing Induced Fit and Conformational Selection With Stopped‐Flow Mixing Experiments

**FIGURE 3:**
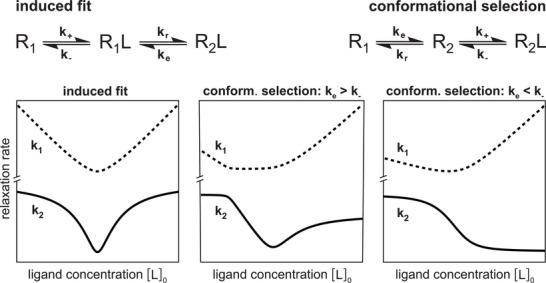
Characteristic relaxation rates $k_{1}$ and $k_{2}$ of the induced‐fit and conformational‐selection model as functions of the ligand concentration [L]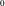 in mixing experiments [22]. In the induced‐fit model, $k_{1}$ and $k_{2}$ are symmetric functions around a minimum located at ${\left[L\right]}_{0}^{\min}={\left[P\right]}_{0}-K_{D}$ where ${\left[P\right]}_{0}$ is the protein concentration in the mixture and $K_{D}=k_{-}k_{e}/\left(k_{+}\left(k_{e}+k_{r}\right)\right)$ is the dissociation constant in the induced‐fit model. In the conformational‐selection model, the smaller relaxation rate $k_{2}$, which dominates the final relaxation into equilibrium, monotonously decreases with [L]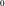 if the conformational excitation rate $k_{e}$ for the transition from $R_{1}$ to $R_{2}$ is smaller than the dissociation rate $k_{-}$ of the complex $R_{2}L$. If $k_{e}$ is larger than $k_{-}$, the relaxation rate $k_{2}$ exhibits a minimum at ${\left[L\right]}_{0}^{\min}≃{\left[P\right]}_{0}\left(k_{e}+k_{-}\right)/\left(k_{e}-k_{-}\right)-K_{D}$ with dissociation constant $K_{D}=k_{-}\left(k_{e}+k_{r}\right)/\left(k_{+}k_{e}\right)$ in the conformational‐selection model, without the symmetry of the induced‐fit model. The relaxation rates $k_{1}$ and $k_{2}$ here have been calculated for the 3‐state pathways from $R_{1}$ to $R_{2}L$ in induced fit and conformational selection, because the rates are not affected by the additional “off‐pathway” conformational exchange in the 4‐state models of Figure 2 if the sum of the rates for this exchange is larger than $k_{1}$ and $k_{2}$, which is plausible for a typically fast conformational relaxation from the minor to the dominant state in this off‐pathway exchange.

Stopped‐flow mixing experiments probe the chemical relaxation into binding equilibrium. In these experiments, two solutions containing the two binding partners are injected into a mixing chamber, and the binding reaction is monitored in the chamber after stopping the injection flow. For both induced‐fit and conformational‐selection binding processes, the final relaxation into binding equilibrium can be described as a double‐exponential relaxation with two relaxation rates, $k_{1}$ and $k_{2}$ [22]. But the two binding mechanisms differ in how $k_{1}$ and $k_{2}$ depend on the ligand concentration [L]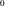 (see Figure 3), which can be used to distinguish induced fit and conformational selection. For an induced‐fit binding process, the functions $k_{1}\left({\left[L\right]}_{0}\right)$ and $k_{2}\left({\left[L\right]}_{0}\right)$ are symmetric around a minimum. For a conformational‐selection process, in contrast, $k_{1}\left({\left[L\right]}_{0}\right)$ and $k_{2}\left({\left[L\right]}_{0}\right)$ do not exhibit this symmetry: the smaller rate $k_{2}$ either decreases monotonously with ${\left[L\right]}_{0}$ if the conformational excitation rate $k_{e}$ of the process is smaller than the dissociation rate $k_{-}$, or exhibits a minimum between asymmetric arms for $k_{e}>k_{-}$ (see Figure 3).

A monotonous decrease of $k_{2}\left({\left[L\right]}_{0}\right)$ thus can only occur for conformational selection, which has been used to identify this binding mechanism in protein systems with $k_{e}<k_{-}$ [23, 24]. Conformational selection has also been identified for a system with $k_{e}>k_{-}$ from an asymmetry of the two arms of the function $k_{2}$ ${\left[L\right]}_{0}$ left and right of the minimum [25]. For these protein systems, only the smaller rate $k_{2}$, also termed $k_{\mathrm{obs}}$, could be observed (i.e., deduced from fits of the stopped‐flow relaxation curves) at the different ligand concentrations [L]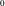.

Induced fit, in contrast, is in general more difficult to identify based on stopped‐flow mixing experiments, because a (near) symmetry of the function $k_{2}\left({\left[L\right]}_{0}\right)$ can also occur for conformational selection. Deducing induced fit from stopped‐flow experiments therefore requires a closer look at the rate constants of the induced‐fit and conformational‐selection models that are obtained from fitting these models to the stopped‐flow data. In this model fitting, the induced‐fit and conformational‐selection mechanisms are simplified from the 4‐state models of Figure 2 to the 3‐state models in Figure 3 to reduce the number of fit parameters. The rates $k_{1}$ and $k_{2}$ are not affected by the additional “off‐pathway” conformational exchange in the 4‐state models if the sum of the rates for this exchange is larger than $k_{1}$ and $k_{2}$, which is plausible for a typically fast conformational relaxation from the minor to the dominant state in this off‐pathway exchange.

## 4 Induced fit of the Neurotensin Receptor 1 Inferred From Stopped‐Flow Mixing and Saturation Transfer Difference NMR Experiments

**FIGURE 4:**
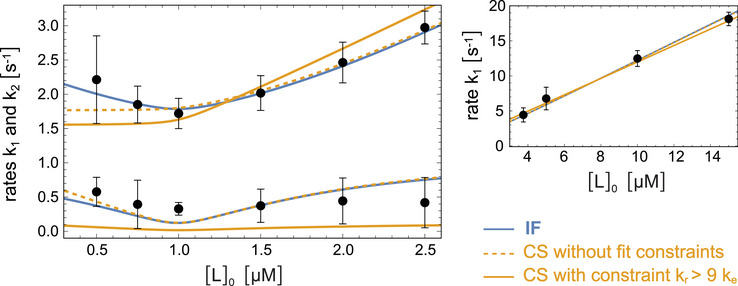
Fits of the relaxation rates $k_{1}$ and $k_{2}$ from stopped‐flow mixing experiments of the thermostabilized receptor NTS1 and the peptide ligand neurotensin (data points) with the induced‐fit (IF) and conformational‐selection (CS) models (colored lines) [26]. For neurotensin concentrations [L]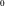 between 0.5 and 2.5 μM, both rates $k_{1}$ and $k_{2}$ could be determined from double‐exponential fits of the stopped‐flow relaxation curves. For neurotensin concentrations [L]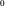 between 3.75 and 15 μM, the rate $k_{1}$ was determined from single‐exponential fits of the initial relaxation in the stopped‐flow experiments. The NTS1 concentration in all mixing experiments was [P]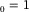 μM. All data points were jointly fitted with both the induced‐fit and conformational‐selection with the three rate constants $k_{e}$, $k_{r}$, and $k_{-}$ as fit parameters. The fourth rate constant of the models, $k_{+}$ was replaced by the experimentally measured dissociation constant $K_{D}=6\pm 2$ nM. The resulting fit values are shown in Table 1. For the conformational‐selection model, both an unconstrained fit and a fit with the constraints $k_{r}>9k_{e}$ was performed. In this constraint fit, the relative probability of $R_{2}$ in the unbound state is limited to plausible values smaller than 10% (see text). The goodness of the fits assessed with the standard Akaike Information Criterion (AIC) indicates that the constraint fit is poor with an AIC value of 33.8 that is significantly larger than the AIC value 15.0 for the unconstrained fit of the conformational‐selection model and the nearly equal AIC value of 15.1 for the fit of the induced‐fit model. The figure is adapted from Ref. 26.

The neurotensin receptor 1 (NTS1) is a GPCR that is activated by the endogenous peptide neurotensin as ligand [27]. NTS1 is primarily expressed in the central nervous system and gastrointestinal tract [28] and regulates neurological processes including dopamine transmission and GABAergic system modulation [29]. For a thermostabilized NTS1 variant solubilized in detergent as replacement for the native membrane environment, stopped‐flow mixing experiments allowed to determine the two binding relaxation rates $k_{1}$ and $k_{2}$ at concentrations [L]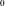 of the ligand neurotensin between 0.5 and 2.5 μM and the relaxation rate $k_{1}$ at additional concentrations [L]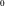 between 3.75 and 15 μM [26] (see data points in Figure 4). The protein concentration in all mixing experiments was [P]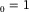 μM, and the relaxation into binding equilibrium in the mixing chamber was monitored via the increase of the intrinsic tryptophan fluorescence of the protein upon peptide binding. The stopped‐flow mixing experiments were conducted in the absence of the associated G protein, because the G protein did not induce any further changes to the conformational dynamics of the NTS1‐neurotensin complex in NMR experiments [26]. Neurotensin alone thus appears to sufficiently stabilize the active conformation of thermostabilized NTS1.

To deduce the binding mechanism from the stopped‐flow data, the functions $k_{1}\left({\left[L\right]}_{0}\right)$ and $k_{2}\left({\left[L\right]}_{0}\right)$ for the induced‐fit and conformational‐selection model were fitted to the data with the three rate constants $k_{e}$, $k_{r}$, and $k_{-}$ as fit parameters (see colored lines in in Figure 4). The fourth rate constant of the models, $k_{+}$, was replaced by the experimentally measured dissociation constant $K_{D}=6\pm 2$ nM of the NTS1‐neurotensin complex, which depends on the rate constants of the models (see caption of Figure 3). In the induced‐fit model, $k_{e}$ and $k_{r}$ are the conformational transition rates between $R_{1}L$ and $R_{2}L$, and $k_{-}$ is the dissociation rate of $R_{1}L$. In the conformational‐selection model, $k_{e}$ and $k_{r}$ denote the transition rates between $R_{1}$ and $R_{2}$, and $k_{-}$ is the dissociation rate of $R_{2}L$.

Fitting the conformational‐selection model without constraints on rate parameters leads to a large probability $P\left(R_{2}\right)=k_{e}/\left(k_{e}+k_{r}\right)>50$% of the active conformation $R_{2}$ in the unbound state (see Table 1), in contradiction to X‐ray crystal structures of NTS1 [30] and to NMR data of thermostabilized NTS1 [26, 31], which indicate that the inactive conformation is the dominant conformation in the unbound states of both NTS1 and the thermostabilized NTS1 variant used in the stopped‐flow experiments. Fits in which $P(R_{2})$ is constrained to plausible values < 10% (i.e., to rate parameters $k_{r}>9k_{e}$) poorly match the stopped‐flow data (see Figure 4). With the induced‐fit model, in contrast, the stopped‐flow data can be well fitted with plausible excitation and relaxation rate constants $k_{e}$ and $k_{r}$ for the conformational exchange between $R_{1}L$ and $R_{2}L$, and with a dissociation rate constant $k_{-}$ of the bound excited state $R_{1}L$ of about 0.6 $s^{-1}$. Moreover, the conformational exchange rate constants $k_{e}$ and $k_{r}$ obtained from the stopped‐flow data in the induced‐fit model are in good agreement with the exchange rate constants 0.08 and 1.23 $s^{-1}$ measured in saturation transfer difference (STD) NMR experiments of NTS1 bound to a fluorinated neurotensin variant. This agreement of conformational exchange rates in the bound state obtained from distinct experiments is a rather strong indication of induced fit as the binding mechanism.

**TABLE 1:** Values of rate constants for the induced‐fit (IF) and conformational‐selection (CS) model obtained from the fits in Figure 4 in units of $s^{-1}$.

fitted model	$k_{e}$	$k_{r}$	$k_{-}$
IF	0.015 [0, 0.04]	1.1 [0.7, 1.6]	0.6 [0.25, 0.95]
CS without constraints	1.2 [0.7, 1.7]	0.6 [0.2, 1.0]	0.005 [0.002, 0.009]
CS with constraint $k_{r}>9k_{e}$	0.16 [0, 0.8]	1.4 [0.9, 1.9]	0.001 [0, 0.004]

*Note*: Numbers in brackets indicate 95% confidence intervals.

## 5 Induced Fit of the $\beta_{2}$‐Adrenergic Receptor Inferred From a Decrease of Ligand Association Rates After Stabilization of the Active Receptor Conformation

The $\beta_{2}$‐adrenergic receptor ($\beta_{2}\mathrm{AR}$) is a prototypic GPCR that recognizes epinephrine (adrenaline) as ligand and mediates a variety of physiological responses, including smooth muscle relaxation and bronchodilation [33]. To investigate the interplay between ligand binding on the extracellular side and G‐protein binding on the intracellular side of $\beta_{2}\mathrm{AR}$, Devree et al. [32] monitored the association kinetics of ligands to $\beta_{2}\mathrm{AR}$‐G protein complexes. In these complexes, the G protein is either bound to the nucleotide GDP, or nucleotide‐free.

For the nucleotide‐free G protein in complex with $\beta_{2}\mathrm{AR}$, Devree et al. [32] observed significantly reduced ligand association rates compared to the GDP‐bound G protein. A significant reduction in ligand association rates was also observed for $\beta_{2}\mathrm{AR}$ bound to the nanobody Nb80, which stabilizes the active $\beta_{2}\mathrm{AR}$ conformation [34]. From the similar effect of Nb80 and nucleotide‐free G protein on ligand association rates, Devree et al. [32] concluded that both stabilize the active $\beta_{2}\mathrm{AR}$ conformation, and that ligand binding is impaired in this conformation.

**FIGURE 5:**
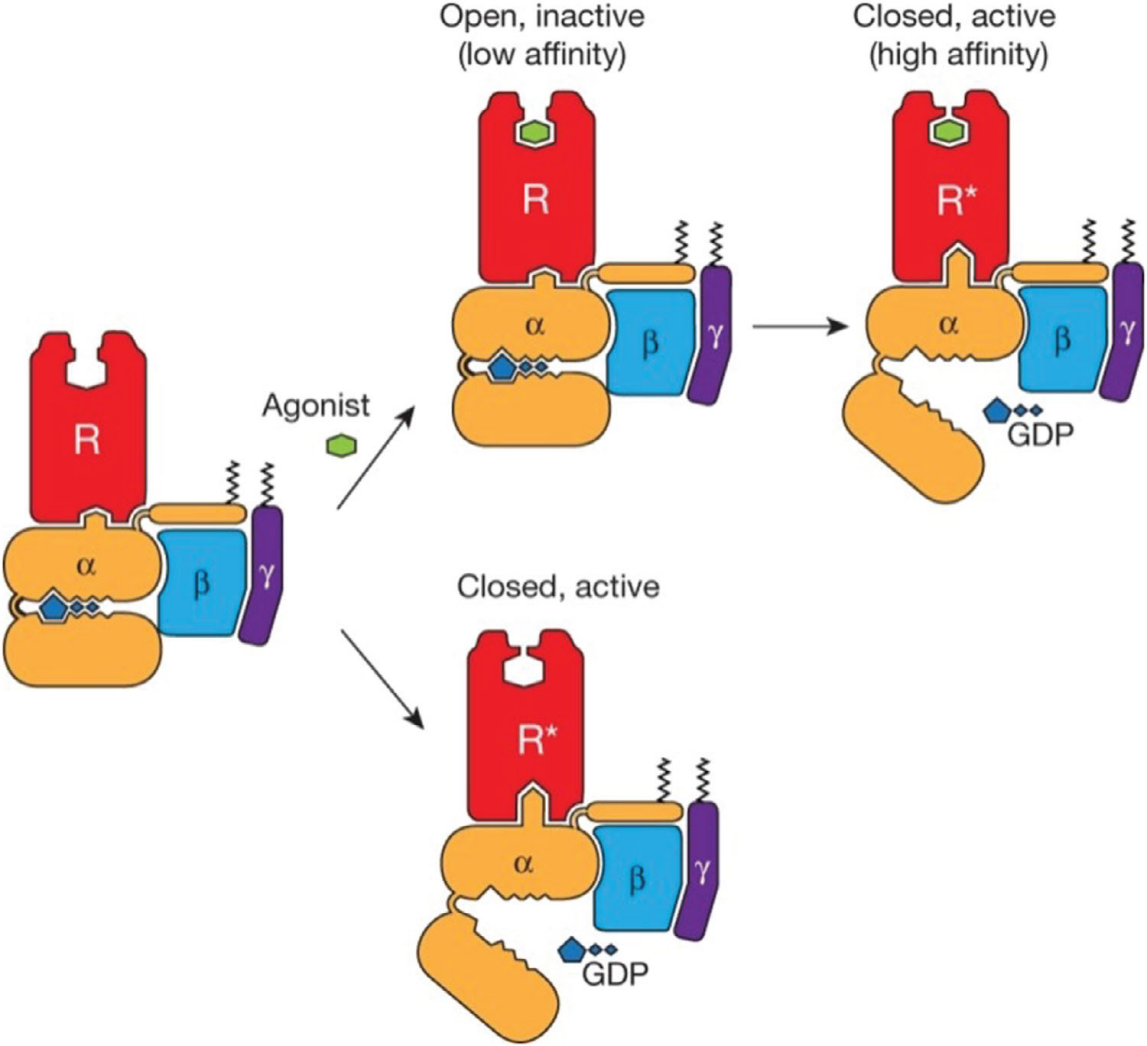
Kinetic mechanism for $\beta_{2}\mathrm{AR}$ activation [32]. The G protein is represented as heterotrimer with subunits α, β, and γ. The G protein with GDP‐bound α‐subunit is associated with the inactive, open $\beta_{2}\mathrm{AR}$, and the nucleotide‐free G protein is associated with the active, closed $\beta_{2}\mathrm{AR}$ conformation. Binding of ligand agonists only occurs in the inactive $\beta_{2}\mathrm{AR}$ conformation in this mechanism. The figure is reproduced with permission from Ref. 32.

In the kinetic mechanism for $\beta_{2}\mathrm{AR}$ activation suggested by Devree et al. [32] (see Figure 5), the nucleotide‐free G protein is associated with the active $\beta_{2}\mathrm{AR}$ conformation, while the GDP‐bound G protein is associated with the inactive $\beta_{2}\mathrm{AR}$ conformation, which is the ligand‐binding‐competent conformation in this mechanism. The significantly reduced ligand association rates of $\beta_{2}\mathrm{AR}$ in complex with the nucleotide‐free G protein can be understood from the stabilization of the active conformation in this complex. This kinetic mechanism is the induced‐fit mechanism of Figure 2 with $R_{1}$ corresponding to the inactive $\beta_{2}\mathrm{AR}$ conformation in complex with the GDP‐bound G protein, and $R_{2}$ corresponding to the active $\beta_{2}\mathrm{AR}$ conformation in complex with the nucleotide‐free G protein.

As structural explanation for impaired ligand binding in the active $\beta_{2}\mathrm{AR}$ conformation, Devree et al. [32] point out a lid‐like structure formed by two aromatic residues that closes over the ligand‐binding site in this conformation. Mutating one of these bulky aromatic residues to the small residue alanine diminishes the effect of the nanobody Nb80 on ligand association rates, which supports the lidding effect of these residues [32].

## 6 Conclusions

The kinetic and structural evidence revisited here indicates that induced fit is the kinetic allosteric mechanism of the small‐molecule‐activated GPCR $\beta_{2}\mathrm{AR}$ and of the peptide‐activated GPCR NTS1. Based on structural similarities, induced fit likely is the allosteric mechanism also of other small‐molecule‐activated and peptide‐activated GPCRs. Constriction and closing of the ligand‐binding site in the active conformation is rather common for small‐molecule‐activated and peptide‐activated GPCRs and likely prevents binding in the active conformation. Similar to $\beta_{2}\mathrm{AR}$, the active structure of the muscarinic acetylcholine receptor exhibits a lid‐like structure over the ligand‐binding site [32, 35]. For this receptor as well as for the μ‐opioid receptor, Devree et al. [32] reported a decrease of ligand association rates after stabilization of the active conformation akin to $\beta_{2}\mathrm{AR}$, which indicates that the induced‐fit allosteric mechanism of Figure 5 is shared by the receptors. Also, for the P2Y12 receptor, a lid‐like structure over the ligand binding site has been observed for a complex with a close analogue of the endogenous ligand ADP [36].

Compared to small‐molecule‐activated GPCRs, the binding sites of peptide‐activated GPCRs tend to be larger and more open to accommodate the peptide ligands. However, structural data indicate a constriction of the binding site in the active conformation, leading to tight interactions between receptor and activating, agonist peptides [30, 37, 38, 39, 40]. This binding‐site constriction provides a structural explanation for the induced‐fit binding of NTS1 to neurotensin [26], and lends plausibility to induced fit as general allosteric mechanism of peptide‐activated GPCRs. Non‐activating inverse agonists  of NTS1, in contrast, have been found to induce a widening of the ligand‐binding pocket [30], which likely prevents the change to the activated conformation of NTS1 and, thus, provides a structural explanation for the inverse agonism.

The induced‐fit binding model of GPCRs in Figure 2 is compatible with the existence of several inactive or active conformations as long as the conformational exchanges within the ensembles of inactive and active conformations are fast compared to the exchange between inactive and active conformations. For $\beta_{2}\mathrm{AR}$, such a fast exchange between two inactive conformations has been observed in NMR experiments [41, 42]. In the kinetic models of Figure 2, the focus is on the slowest, rate‐limiting conformational transitions that dominate the relaxation into binding equilibrium and the exchange between bound and unbound states. In the induced‐fit model, the effective ligand off‐rate $k_{\text{off}}=k_{e}k_{-}/\left(k_{r}+k_{-}\right)$ depends on the conformational transition rates $k_{e}$ and $k_{r}$ between the bound states $R_{1}L$ and $R_{2}L$ and on the unbinding rate $k_{-}$ of the ligand from the inactive bound state $R_{1}L$ [10, 20]. For the rates in Table 1 obtained from fits of the stopped‐flow data for thermostabilized NTS1, the ligand off‐rate is close to the conformational transition rate $k_{e}$ for the change from the active bound state $R_{2}L$ to the inactive state $R_{1}L$ and, thus, limited by $k_{e}$. Agents that directly or indirectly stabilize the active conformation of NTS1, thus increase the residence time of the bound ligand. In the conformational‐selection model, in contrast, the effective off‐rates are typically limited by the unbinding rate $k_{-}$ of the ligand from the predominantly populated bound state $R_{2}L$ [10].

## Conflicts of Interest

There are no conflicts to declare.

## Data Availability

Data sharing is not applicable to this article as no datasets were generated during the current study.
